# Heat exposure and self-rated health in older Chinese adults: the mediating roles of chronic disease and intergenerational support, 2008–2018 CLHLS

**DOI:** 10.3389/fpubh.2025.1636724

**Published:** 2025-09-25

**Authors:** Huan Wang, Danyang Wang

**Affiliations:** Institute of Population Research, Hohai University, Nanjing, China

**Keywords:** climate change, heat exposure, China, older adults, self-rated health, mechanism paths

## Abstract

**Background:**

Under the dual pressures of global warming and accelerated population aging, rising temperatures pose a particularly serious threat to the older population. However, systematic evidence on the heat exposure-response pathway is still scarce. This study aims to explore the effects of heat exposure on self-rated health and its pathways in older adults in China. We predicted that heat exposure would reduce the self-rated health of older adults, and that chronic diseases and intergenerational support would mediate this effect.

**Methods:**

We linked health data from 9,670 participants in the Chinese Longitudinal Healthy Longevity Survey (CLHLS, 2008–2018 waves) with meteorological records from the National Climatic Data Center (NCDC). Individual fixed-effects models were employed to disentangle acute versus cumulative thermal effects, while Bootstrap-mediated path analysis quantified the mediating mechanisms involving chronic disease proliferation and deterioration of intergenerational support.

**Results:**

Heat exposure has a time effect on the health risk of older adults, and long-term heat exposure (*β* = −0.156, *p* < 0.01; *β* = −0.003, *p* < 0.01) significantly reduces self-rated health through the cumulative effect of health disadvantages than short-term exposure (*β* = 0.004, *p* < 0.1; *β* = −0.001, *p* > 0.1). The increase in the number of chronic diseases (*β* = 0.260, *p* < 0.05) and the weakening of children’s intergenerational support (*β* = −0.052, *p* < 0.01; *β* = −0.023, *p* < 0.01) constitute a mediating pathway at individual and household level separately.

**Conclusion:**

We found that chronic diseases and intergenerational support from children mediated the effect of heat exposure on the deterioration of self-rated health in older adults. Empirical evidence substantiates the necessity for a tiered intervention framework encompassing: individual-level chronic disease co-management protocols; household-driven initiatives to reinforce intergenerational support. This stratified approach alleviates bioclimatic risks through coordinated physiological adaptation and optimization of kinship network.

## 1 Introduction

The Intergovernmental Panel on Climate Change (IPCC) projects a global temperature increase ranging from 3.3 °C to 5.7 °C by the end of the 21st century (1). With the intensification of global climate change, the frequency, intensity, and duration of extreme heat events have significantly increased (2, 3), pose a significant threat to human health. Over the past two decades, heat-related deaths among vulnerable populations worldwide have increased by 53.7%, with 296,000 individuals succumbing to high temperatures in 2018 alone (4). This phenomenon, compounded by the accelerating pace of population aging, is expected to result in 246 million people aged 65 and above being exposed to dangerous high temperatures by 2050, with a particularly pronounced proportion in Asia and Africa (5). Concurrently, historical evidence demonstrates that the older population, as a vulnerable group under climate change, face multidimensional health risks, necessitating systematic research to elucidate the underlying mechanisms (6–11).

Previous studies have revealed the acute effects of heat exposure on mortality rates among older adults (12–15). Notably, research has revealed that older adults’ thermoregulatory capacity is significantly diminished (16). Exposure to high temperatures directly increases their vulnerability to heatstroke and elevates cardiovascular and cerebrovascular risks (17–19). Furthermore, it indirectly contributes to respiratory disease risks through increased atmospheric particulate matter concentrations (16, 20). Additionally, some research has identified a strong correlation between heat exposure and depressive disorders as well as other mental health issues among older adults (21–23).

However, despite the established evidence on various health impacts of heat exposure, existing studies exhibit notable limitations in their approach to assessment. While research has documented specific effects on mortality, physiological conditions, and mental health (24), there remains a lack of comprehensive evaluation that captures the multifaceted nature of heat’s impact on overall health status. Self-rated health, which has been validated as a more holistic indicator of individual health status, offers potential advantages in this context (25). For instance, Lynch demonstrated the utility of self-rated health measures in capturing the long-term health implications of educational disruption among middle-aged and older adults, highlighting its comprehensive nature, predictive validity, and sensitivity (26). However, this valuable metric has been underutilized in research examining the relationship between extreme weather events and health outcomes (27, 28), this limits our understanding of the combined health burden caused by heat exposure.

Additionally, another significant limit in current research lies in its narrow focus on individual-level physiological vulnerability factors, while neglecting the potential protective role of household-level adaptive support mechanisms (29–31). This oversight is particularly noteworthy given that intergenerational support from adult children has been shown to provide crucial emotional, financial, and caregiving assistance during adverse experiences, potentially mitigating various health risks faced by older adults (32, 33). Although Silverstein’s research has demonstrated the vital importance of such intergenerational support for the health outcomes of older adults, especially in challenging circumstances like intensive caregiving needs and widowhood (34, 35), its specific role in protecting against heat exposure risks remains largely unexplored. Given the projected increase in older populations exposed to dangerous temperatures and the documented health impacts of heat exposure, understanding these potential protective mechanisms warrants urgent investigation.

The interaction of a growing climate-vulnerable older population in China with support systems embedded in a culture of filial piety provides an ideal scenario for revealing the association between heat exposure and health in older adults. Firstly, China is a sensitive and significant area of global climate change. From 1961 to 2023, the frequency of extreme high temperature events in China will show a significant increase trend. In 2023, a total of 838 station days of extreme high temperature events occurred in China, 329 more than the normal value (36). Secondly, population aging has become an important trend of China’s population change and a long-term basic national condition (37). By the end of 2023, there will be 290 million people aged 60 years and above in China, accounting for 21.1% of the total population (38), and it is expected to peak around 2050, with an estimated population of about 580 million (39). This large aging cohort significantly increases the population exposed to and vulnerable to climate-related health risks. Thirdly, filial piety culture is a traditional cultural norm in China, which emphasizes the “responsibility of support” of children in return for the “kindness of upbringing” of their parents (40). Intergenerational support may be an important support for older adults in China to cope with environmental stress and serve as a channel for their health protection during high temperature exposure.

In order to capture the dynamic changes in the health status of older adults and the long-term effect of heat exposure, we explored the association between heat exposure and the health of older adults, as well as the role of individual physiological vulnerability and intergenerational support of children in this relationship, based on the data of the Chinese Longitudinal Health and Longevity Survey (CLHLS) from 2008 to 2018. Based on the existing studies, we proposed hypothesis H1: heat exposure would reduce the self-rated health of older adults. Secondly, based on the physiological vulnerability theory of older adults, heat exposure may trigger the adverse effects of chronic diseases, thereby reducing the health level. We proposed hypothesis H2: Chronic diseases play a mediating role in the reduction of self-rated health in older adults by heat exposure. In addition, based on the protective effect of intergenerational support of children on the health risk faced by older adults during the period of heat exposure, we proposed hypothesis H3: intergenerational support plays a mediating role in the reduction of self-rated health of older adults during the period of heat exposure.

## 2 Methods

### 2.1 Data sources

The meteorological data utilized in this study were obtained from the National Climatic Data Center (NCDC), a division of the National Oceanic and Atmospheric Administration. The dataset encompasses observations from over 400 meteorological stations across China, encompassing key climatic variables including temperature, atmospheric pressure, dew point, wind direction and speed, cloud cover, and precipitation.

The data on older adults were derived from the Chinese Longitudinal Healthy Longevity Survey (CLHLS)^1^, conducted by the Center for Healthy Aging and Development at Peking University. This study employs longitudinal tracking data spanning the period from 2008 to 2018, encompassing 23 provinces and municipalities directly under the central government across China, which collectively represent the eastern, central, and western regions of the country. During the 2008–2018 period, the CLHLS administered four waves of follow-up surveys, with an initial sample size of 16,954. After excluding deceased individuals and those lost to follow-up, a total of 2,440 surviving participants were retained for analysis.

### 2.2 Outcome assessment

The dependent variable is the self-rated health of older adults. Based on the question, “How do you perceive your current health status?” Responses ranging from “Very unhealthy” to “Very healthy” were assigned values from 1 to 5, creating a continuous variable with higher values indicating better health status.

### 2.3 Heat exposure assessment

The independent variable is the measure of heat exposure. Considering that temperature has a lagged effect on health (41), both short-term and long-term heat exposure were used as proxy variables for high-temperature indicators. Short-term heat exposure was measured using the daily maximum temperature and the heat index. The heat index (HI) is a comprehensive indicator that combines air temperature and relative humidity to more accurately reflect the perceived temperature by the human body (42). In this paper, the daily maximum temperature (T) and relative humidity (RH) are used for calculation, which can assess the health risks during the hottest period of the day (43). The detailed formula is shown in Equation 1.


(1) $$\begin{array}{l} \mathrm{HI}=-42.379+2.04901523T+10.14333127\mathrm{RH}\\ -0.22475541\mathrm{TRH}-6.83783\cdot 10^{-3}T^{2}\\ -5.481717\cdot 10^{-2}{\mathrm{RH}}^{2}+1.22874\cdot 10^{-3}T^{2}\mathrm{RH}\\ +8.5282\cdot 10^{-4}{\mathrm{TRH}}^{2}-1.99\cdot 10^{-6}T^{2}{\mathrm{RH}}^{2} \end{array}$$

Long-term heat exposure was measured using the annual average-temperature and annual hot days. The annual hot days was derived based on the calculation of the Excess Heat Factor (EHF). The EHF can dynamically calibrate historical climate benchmarks with the current level of heat adaptation, and is thus capable of quantifying the cumulative impact of the intensity and duration of heat exposure. Previous studies have shown that this indicator can effectively assess the long-term health risks associated with heat exposure (44). The EHF was designed by Perkins and is composed of two main components: EHI_sig_ and EHI_accl_ (45). EHI_sig_ indicates whether the average temperature over the most recent 3 days at a location exceeds the long-term climatological extreme high temperature (T95) for that location. T95 is commonly represented by the 95th percentile value of the 30-year daily average temperatures arranged in ascending order for that station. EHI_accl_ indicates whether the average temperature over the most recent 3 days exceeds the average temperature over the preceding 3–32 days at that location. The detailed formula is shown in Equations 2–4.

For the purpose of this study, a high-temperature event is defined as occurring when EHF > 0. The cumulative number of high-temperature events in a year constitutes the annual hot days. Therefore, EHF is a non-negative number that exceeds both long-term and recent extreme temperature thresholds. Equation 4 can thus be rewritten as Equation 5.


(2) $$\mathrm{EH}I_{\mathrm{sig}}=\frac{T_{i}+T_{i-1}+T_{i-2}}{3}-T_{95}$$

(3) $$\mathrm{EH}I_{\text{accl}}=\frac{T_{i}+T_{i-1}+T_{i-2}}{3}-\frac{T_{i-3}+,\ldots ,+T_{i-32}}{30}$$

(4) $$\mathrm{EHF}=\mathrm{EH}I_{\mathrm{sig}}\times \max(1,\mathrm{EH}I_{\text{accl}})$$

(5) $$\mathrm{EHF}=\max(0,{\mathrm{EHI}}_{\mathrm{sig}})\times \max(1,\mathrm{EH}I_{\text{accl}})$$

### 2.4 Mediator variable

This study includes chronic diseases as individual-level mechanism variables, and intergenerational support as a household-level mechanism variable. “Chronic diseases” are measured based on the question, “Do you currently suffer from any of the following chronic diseases (25 types)?” The number of chronic diseases reported is summed. Intergenerational support is assessed across three dimensions: economic support, care support, and emotional support from children to parents. Economic support and care support are dichotomized based on whether cash is provided by (grand) children or their spouses and whether care is provided for older adults (1 = yes, 0 = no). Emotional support is measured by the questions, “Who do you talk to the most?” and “If you have worries or thoughts, who do you talk to first?” If the respondent answers that it is their (grand) children or their spouses for at least one of the questions, the variable is set to 1; otherwise, it is 0.

### 2.5 Covariates

Referring to previous studies on factors influencing the health of older adults, this study controls for variables related to demographic characteristics, socioeconomic characteristics, and physical health status. Demographic characteristics include gender, age, place of residence, and marital status. Socioeconomic characteristics include education, occupation, cohabitation type, and living standards. The physical condition of older adults is analyzed based on their activities of daily living (ADL) and life satisfaction. Specific variable coding is shown in Table 1.

**Table 1:** Descriptive statistics of variables.

Variable Types	Variables	Units and Values	Percentage/Mean (S. D.)
Dependent Variable	Self-rated Health	1 = very unhealthy	3.39 (0.10)
		2 = unhealthy	
		3 = average	
		4 = healthy	
		5 = very healthy	
Independent Variables	Short-term Heat Exposure		
	Daily Max-temperature	Unit: Celsius	28.05 (7.25)
	Heat Index	Unit: Fahrenheit	100.05 (20.08)
	Long-term Heat Exposure		
	Annual Avg-temperature	Unit: Celsius	17.06 (3.84)
	Annual Hot Days	Unit: Days/Year	20.79 (12.04)
Control Variables	Gender	0 = female; 1 = male	46.68%
	Age	0 = aged 60–79; 1 = aged 80 and above	45.64%
	Residence	0 = rural; 1 = urban	47.41%
	Marital Status	0 = unmarried; 1 = married	49.78%
	Education Level	0 = uneducated; 1 = educated	52.13%
	Pre-60 Occupation Types	0 = high-income occupation; 1 = low-to-middle income occupation	91.39%
	Co-residence Types	0 = not living with family; 1 = living with family	78.28%
	Living Standards	1 = very difficult	3.01 (0.68)
		2 = difficult	
		3 = average	
		4 = comfortable	
		5 = very comfortable	
	ADL	Reverse Scoring: 14–45 points	17.83 (6.03)
	Life Satisfaction	1 = very dissatisfied	3.68 (0.95)
		2 = dissatisfied	
		3 = average	
		4 = satisfied	
		5 = very satisfied	
	Chronic Diseases	Unit: Type/Person	1.20 (1.38)
	Intergenerational Support		
	Economic Support	0 = none; 1 = yes	75.66%
	Care Support	0 = none; 1 = yes	22.69%
	Emotional Support	0 = none; 1 = yes	79.77%

S.D. = Standard deviation; Standard deviation in parentheses.

### 2.6 Statistical models

To accurately assess the impact of heat exposure on health, this study employs the Fixed Effects Model (FEM) for analysis. The FEM effectively utilizes the individual variation information within time-series data and controls for individual characteristics that are invariant over time but may confound the results (46). We analyzed older adults’ self-rated health data and daily temperature tracking records across different time periods using the fixed effects model. This approach enabled us to more accurately measure the health impacts of temperature fluctuations on individuals. The analysis helped establish clear causal relationships between high temperatures and health outcomes in this population. The following Equation 6 is the FEM for assessing the impact of heat exposure on the self-rated health of older adults:


(6) $${\text{Health}}_{\mathrm{it}}=c_{1}\cdot \mathrm{Hea}t_{\mathrm{it}}+\text{Contro}l_{\mathrm{it}}+\mu_{i}+\varepsilon_{\mathrm{it}}$$

Based on the benchmark regression, this study employs the classic “three-step method” and the Bootstrap test to sequentially examine the pathways of individual and household intergenerational support (Equations 7–9).


(7) $${\text{Health}}_{\mathrm{it}}=c_{1}\cdot \mathrm{Hea}t_{\mathrm{it}}+\text{Contro}l_{\mathrm{it}}+\mu_{i}+\varepsilon_{\mathrm{it}}$$

(8) $${\text{Mediation}}_{\mathrm{it}}=a_{1}\cdot \mathrm{Hea}t_{\mathrm{it}}+\text{Contro}l_{\mathrm{it}}+\mu_{i}+\varepsilon_{\mathrm{it}}$$

(9) $${\text{Health}}_{\mathrm{it}}=c_{1}^{\prime }\cdot \mathrm{Hea}t_{\mathrm{it}}+b_{1}\cdot \text{Mediatio}n_{\mathrm{it}}+\text{Contro}l_{\mathrm{it}}+\mu_{i}+\varepsilon_{\mathrm{it}}$$

Note. Health: self-rated health; Heat: short-term heat exposure (daily maximum temperature and heat index), long-term heat exposure (annual average-temperature and annual hot days); Control: Covariates; Mediation: chronic diseases and intergenerational support; $\mu_{i}$: individual fixed effects; $\varepsilon_{\mathrm{it}}$: random error term.

### 2.7 Statistical analysis

The results of the descriptive statistical analysis are presented in Table 1. Regarding the dependent variable, the average self-rated health of older adults is 3.39, indicating that most older adults in the sample perceive their health status as “fair” or “fairly healthy,” reflecting an overall good to moderately high level of health. Among the temperature indicators, the daily maximum temperature is relatively high and exhibits some volatility, while the heat index also shows a high level of heat exposure. In terms of long-term heat exposure, the annual average-temperature is relatively stable, and the average of annual hot days is 20.79, although there are significant regional differences.

In terms of demographic characteristics of the sample, the proportions of gender, urban/rural residence, and marital status are relatively balanced (46.68%, 47.41%, 49.78%), with a slightly lower proportion of older adults in the higher age groups (45.64%). Regarding socio-economic characteristics, the educational level of older adults is generally low (52.13%), and the majority belong to middle-to-low-income occupational groups (91.39%). A higher proportion of older adults live with family members (78.28%), and the living standards are mostly rated as average (mean = 3.01).

In terms of health, the overall activities of daily living among older adults are good, and on average, older adults suffer from at least one chronic disease. Additionally, the life satisfaction score of older adults is skewed toward “fairly satisfied” (mean = 3.68). Regarding intergenerational support, older adults primarily receive economic and emotional support from their children (75.66%, 79.77%), while care support is relatively insufficient (23.26%).

## 3 Results

### 3.1 Association between heat exposure and self-rated health

Prior to conducting the baseline regression, the high-temperature indicators were standardized due to their inconsistent units of measurement. This process aimed to enhance the interpretability of the model and reduce multicollinearity among the variables. These indicators not only capture the immediate effects of daily high temperatures but also quantify the level of annual heat exposure, thereby providing a more comprehensive analysis and comparison of the impacts of short-term and long-term heat exposure on the self-rated health of older adults.

Table 2 reports the impact of heat exposure on the self-rated health of older adults. The results show that the impact of short-term heat exposure is relatively limited, while long-term heat exposure has a more significant negative effect on the health of older adults. In Models M1 and M5, the daily maximum temperature has a significant positive effect on self-rated health (*β* = 0.004, *p* < 0.1; *β* = 0.005, *p* < 0.05), but this effect is not significant in other models. The regression coefficients of the heat index are insignificant across all models, indicating that the direct impact of short-term heat index on self-rated health is weak. In Models M3 to M6, the annual average-temperature shows a significant negative correlation with self-rated health, with a 1 °C increase in annual average-temperature leading to a 0.1–0.15-point decrease in self-rated health among older adults. In Models M3 and M4, the annual hot days is significantly negatively correlated with self-rated health, but this significance diminishes when more control variables are included (*β* = −0.003, *p* < 0.01; *β* = 0.001, *p* > 0.1).

**Table 2:** Regression analysis of the effect of high temperature on self-rated health among older adults.

Variables	M1	M2	M3	M4	M5	M6
Short-term Heat Exposure						
Daily Max-temperature	0.004^*^	−0.000			0.005^**^	0.000
	(0.002)	(0.002)			(0.002)	(0.002)
Heat Index	−0.001	0.001			−0.001	0.000
	(0.001)	(0.001)			(0.001)	(0.001)
Long-term Heat Exposure						
Annual Avg-temperature			−0.156^***^	−0.101^***^	−0.158^***^	−0.100^***^
			(0.026)	(0.022)	(0.026)	(0.022)
Annual Hot Days			−0.003^***^	0.001	−0.003^***^	0.001
			(0.001)	(0.001)	(0.001)	(0.001)
Residence (Rural = 0)		−0.022		−0.019		−0.019
		(0.021)		(0.021)		(0.021)
Age aged 60–79 = 0		−0.105^***^		−0.073^***^		−0.072^**^
		(0.027)		(0.028)		(0.028)
Co-residence Types		0.061^**^		0.054^*^		0.054^*^
(Not Living with Family = 0)		(0.029)		(0.029)		(0.029)
Living Standards		0.123^***^		0.124^***^		0.124^***^
		(0.015)		(0.015)		(0.015)
Marital Status (Unmarried = 0)		0.082^**^		0.070^**^		0.069^**^
		(0.033)		(0.033)		(0.033)
ADL		−0.026^***^		−0.025^***^		−0.025^***^
		(0.002)		(0.002)		(0.002)
Life Satisfaction		0.518^***^		0.518^***^		0.518^***^
		(0.011)		(0.010)		(0.011)
Chronic Disease		−0.080^***^		−0.080^***^		−0.080^***^
		(0.008)		(0.008)		(0.008)
Intergenerational Support						
Economic Support (None = 0)		0.056^**^		0.052^**^		0.052^**^
		(0.022)		(0.022)		(0.022)
Care Support (None = 0)		−0.004		−0.004		−0.004
		(0.023)		(0.023)		(0.023)
Emotional Support (None = 0)		0.079^***^		0.083^***^		0.082^***^
		(0.023)		(0.023)		(0.023)
Constant	3.394^***^	1.493^***^	6.111^***^	3.218^***^	6.104^***^	3.160^***^
	(0.066)	(0.083)	(0.427)	(0.364)	(0.445)	(0.377)
*R* ^2^	0.001	0.344	0.013	0.346	0.014	0.346

Standard errors are in parentheses; ^*^p < 0.1, ^**^p < 0.05, ^***^p < 0.01.

The analysis of control variables indicates that age, cohabitation type, living standards, marital status, activities of daily living, chronic diseases, and life satisfaction collectively influence the health of older adults, with varying effects under heat exposure. Under heat exposure, older adults in the higher age groups have significantly lower self-rated health than those in the lower age groups. Cohabitating with family members and being married have significant positive effects on health. Improved living conditions and higher life satisfaction also contribute to better health among older adults. Additionally, the level of activities of daily living (*β* = −0.025, *p* < 0.01) and the presence of chronic diseases (*β* = −0.080, *p* < 0.01) are significantly negatively correlated with self-rated health. Regarding intergenerational support, economic and emotional support from children both contribute to higher self-rated health among older adults.

The results of the individual fixed-effects model in Table 2 demonstrate that high temperatures have a temporal effect on the health risks of older adults, with long-term heat exposure posing a more significant health risk. Furthermore, robustness tests using random effects and binary probit and logit fixed-effects models for self-rated health confirm that the main results remain unchanged. Hypothesis H1 was confirmed.

### 3.2 Mediation analysis

The mechanistic testing part aims to identify potential pathways of action. After verifying that heat exposure would reduce the self-rated health of older adults, we followed the logic of mediating effect analysis to test the effects of heat exposure on two mediating variables, namely chronic diseases and intergenerational support. A significant increase in the level of chronic disease or a reduction in the level of intergenerational support by heat exposure would indicate a prerequisite for a mediating effect (specific results can be found in Supplementary Tables 1, 2).

Supplementary Tables 1, 2 indicate that heat exposure significantly affected chronic diseases and intergenerational support levels. At the individual level, long-term heat exposure significantly increased the number of chronic diseases. Annual average- temperature had a significant positive effect on the prevalence of chronic diseases (*β* = 0.260, *p* < 0.05), while the number of annual hot days had a relatively small positive effect on the prevalence of chronic diseases (*β* = 0.042, *p* < 0.01). But overall, long-term heat exposure increases the number of chronic diseases in older adults. Although heat index significantly reduced the number of chronic diseases among older adults, the effect of heat index on self-rated health was not significant in base model regression. Therefore, the prerequisite for the mediating effect of chronic diseases on short-term heat exposure is not established. At the household level, long-term heat exposure significantly reduced economic and emotional support, but increased care support. The increase of annual average-temperature and annual hot days significantly reduced the economic support of children to older adults (*β* = −0.283, *p* < 0.01; *β* = −0.052, *p* < 0.01). The increase of annual hot days significantly reduced children’s emotional support for older adults (*β* = −0.023, *p* < 0.01). The increase of annual average-temperature and annual hot days significantly increased children’s care support for older adults (*β* = 0.142, *p* < 0.01; *β* = 0.045, *p* < 0.01). In summary, the mechanism test showed that chronic diseases and intergenerational support from children might be the mediating factors of long-term heat exposure affecting the self-rated health of older adults.

**Figure 1:**
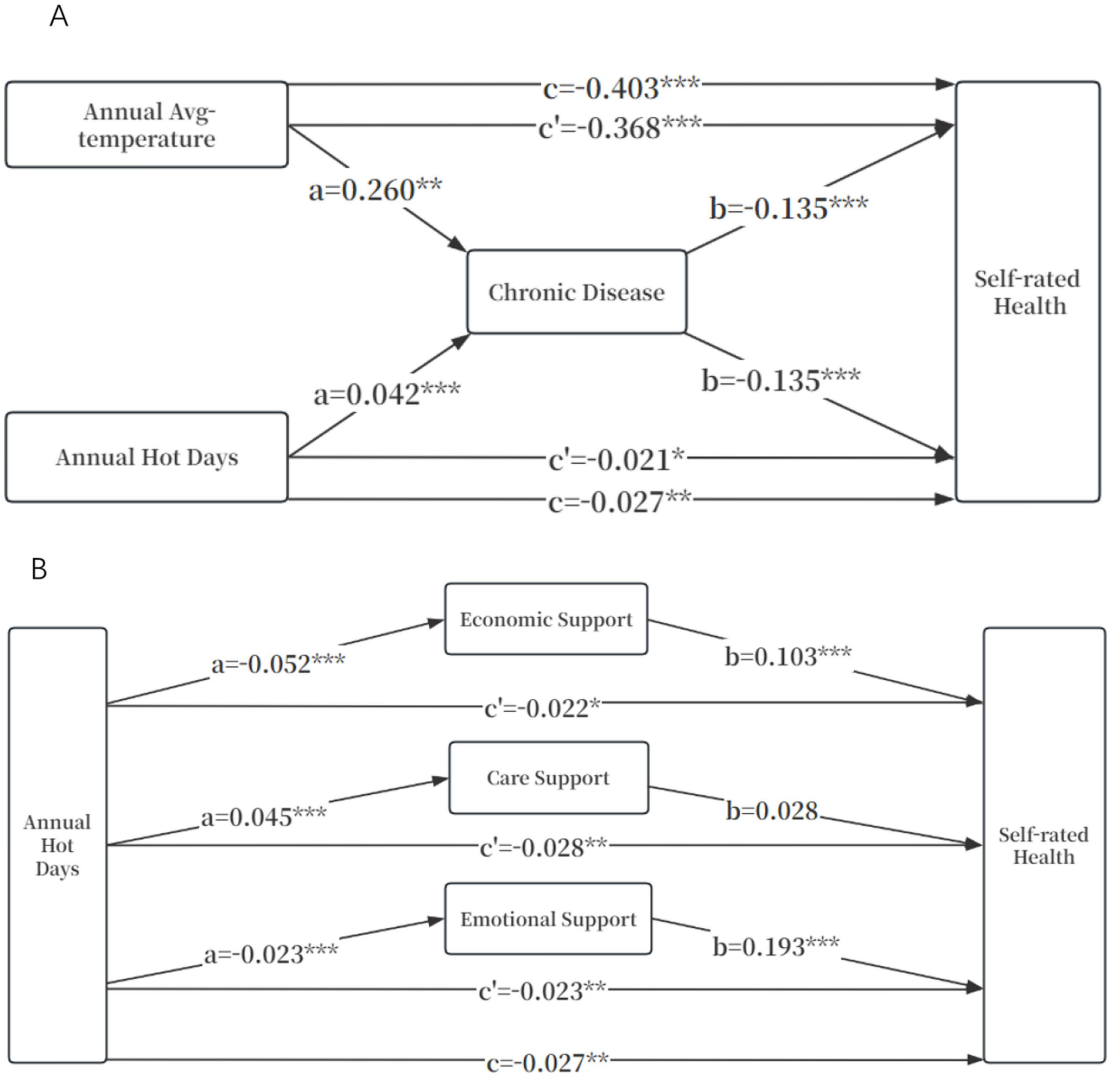
Mediating effect of chronic diseases and intergenerational support between heat exposure and self-rated health. **(A)** Individual-level pathways. **(B)** Household-level pathways. ^*^p < 0.1, ^**^ *p* < 0.05, ^***^ *p* < 0.01; Control variables: Residence, Age, Co-residence type, Living standards, Marital status and ADL. a b c c’ is the path coefficient in the mediating effect analysis of the three-step method.

The construction of the mediation effect triangle diagram in Figure 1 aims to visually demonstrate the complete pathways through which chronic diseases and intergenerational support mediate the impact of heat exposure on self-rated health among older adults, integrating the basic regression and mechanism tests from previous analyses into a unified framework. Results indicate that long-term heat exposure reduces self-rated health levels by significantly increasing chronic disease and reducing economic and emotional support from children.

As shown in Figure 1A, when chronic diseases were not included, the total effect of the annual average-temperature on self-rated health was −0.403 (*p* < 0.01). After including chronic diseases, the direct effect of the annual average-temperature on self-rated health was −0.368 (*p* < 0.01), which was weaker than the total effect, and the mediating effect accounted for approximately 8.71% (0.260 * 0.135/0.403). Similarly, the direct effect of the annual hot days on self-rated health was −0.021 (*p* < 0.1), which was weaker than the total effect (*β* = −0.027; *p* < 0.05), and the mediating effect accounted for approximately 21%. This further supports the partial mediating role of chronic diseases in the long-term exposure to high temperatures on the self-rated health of older adults.

As shown in Figure 1B, when intergenerational support was not included, the total effect of annual hot days on the self-rated health of older adults was −0.027 (*p* < 0.05). Firstly, after including economic support, the direct effect of annual hot days on self- rated health was −0.022 (*p* < 0.1), and the mediating effect accounted for approximately 24.35%; secondly, after including care support, we found that the impact of care support on the self- rated health of older adults was not significant (*β* = 0.028; *p* > 0.1), so the mediating effect of care support was not significant; finally, after including emotional support, the direct effect of annual hot days on self- rated health was −0.023 (*p* < 0.05), and the mediating effect accounted for approximately 19.3%. This suggests that the economic and emotional support from children have some mediating effects in the path through which the increase in annual hot days leads to a decline in the self- rated health of older adults.

Similarly, we also constructed a mediating triangle diagram of the impact of average annual-temperature on self-rated health through economic support and care support (emotional support was excluded after the mechanism test; Supplementary Figure 1). We found that economic support was a partial mediating factor, with the mediating effect accounting for approximately 7.62%. The influence of care support was not significant.

Based on the path relationships revealed by the above-mentioned intermediary triangle diagram, in order to verify the statistical reliability of the mediating effect of chronic diseases and intergenerational support, this study further conducted repeated tests using the Bootstrap sampling method (Table 3). The indirect effect values of chronic diseases on the annual average-temperature and the annual hot days were −0.035 (*p* < 0.01) and −0.006 (*p* < 0.01), respectively; the indirect effect values of economic support on the annual average-temperature and the annual hot days were −0.029 (*p* < 0.1) and −0.005 (*p* < 0.01), respectively; the impact of care support was relatively limited, with the indirect effect values on the annual average-temperature and the annual hot days being 0.004 (*p* < 0.1) and 0.001 (*p* < 0.01), respectively; the indirect effect value of emotional support on the annual hot days was −0.004 (*p* < 0.01). In conclusion, the Bootstrap test confirmed hypotheses H2 and H3. Long-term heat exposure significantly reduced the self-rated health level of older adults through the dual paths of exacerbating chronic diseases and weakening the economic/emotional support from children.

**Table 3:** Bootstrap analysis of the mediating effect of chronic diseases and intergenerational support on the relationship between heat exposure and self-rated health (*N* = 9,760).

Pathways	Effect	Coefficient	Bootstrap standard error	*p*_value
Annual Avg-temperature—Chronic disease—Self-rated Health	Direct effect	−0.368	0.009	0.000
	Indirect effect	−0.035	0.002	0.000
Annual Hot days—Chronic disease—Self-rated Health	Direct effect	−0.021	0.010	0.024
	Indirect effect	−0.006	0.002	0.000
Annual Avg-temperature—Economic support—Self-rated Health	Direct effect	−0.375	0.009	0.000
	Indirect effect	−0.029	0.001	0.060
Annual Hot days—Economic support—Self-rated Health	Direct effect	−0.022	0.010	0.055
	Indirect effect	−0.005	0.001	0.000
Annual Avg-temperature—Care support—Self-rated Health	Direct effect	−0.407	0.009	0.000
	Indirect effect	0.004	0.001	0.087
Annual Hot days—Care support—Self-rated Health	Direct effect	−0.028	0.001	0.003
	Indirect effect	0.001	0.009	0.000
Annual Hot days—Emotional support—Self-rated Health	Direct effect	−0.023	0.010	0.188
	Indirect effect	−0.004	0.001	0.000

Control variables: Residence, Age, Co-residence type, Living standards, Marital status and ADL.

## 4 Discussion

This study elucidates the temporal effects of heat-related health risks and its mediating mechanisms among Chinese older adults, providing novel evidence for advancing theoretical frameworks and policy development regarding climate-health interactions. Departing from previous studies focusing on acute mortality effects (47), our analysis of longitudinal data from China reveals that long-term heat exposure, compared to short-term exposure, not only exacerbates the burden of chronic diseases, consistent with existing research (21, 48), but also significantly undermines intergenerational support systems, which constitute the core pillar of care for older adults in China. These findings provide an empirical foundation for developing stratified interventions within China’s healthy aging strategy to address the compounded challenges of climate change and population aging.

Our findings revealed that heat exposure has a significant time-gradient effect on the health of older adults: short-term heat exposure has a small impact, while long-term heat exposure will accumulate health disadvantages and lead to health deterioration. Thus, the hypothesis H1 that heat exposure would reduce the self-rated health of older adults was confirmed. This finding echoes the physiological adaptation theory proposed by Gronlund. While short-term heat stress may trigger transient thermoregulatory responses such as vasodilation and sweating (49), prolonged exposure overwhelms these mechanisms, consistent with Kenny’s observation of diminishing resilience under chronic thermal strain (50). The longitudinal design captures how repeated heat episodes amplify pre-existing chronic conditions—a pathway recently highlighted in studies of cardiovascular and respiratory morbidity (48, 51). Notably, most studies have focused on the association between high temperatures and mortality (18, 19). However, death is merely the extreme manifestation of declining health. During heat events, the vast majority of older adults individuals experience direct health impairments, such as having a lower self-rated health, whereas only a small minority with severe physical vulnerability face mortality due to their inability to withstand such risks. Therefore, this study revealed that heat exposure significantly reduced self-rated health among older adults through aggravating chronic diseases and weakening intergenerational support, which was a sensitive predictor of mortality risk (52). The impact pathways of self-rated health identified in our study provide a scientific basis for early intervention in the health effects of high temperatures on older adults. In addition, this temporal differentiation challenges homogeneous approaches to heat risk assessment. Unlike research emphasizing acute mortality spikes during heatwaves (47), our focus on self-rated health uncovers subtler, incremental deterioration processes. The erosion of adaptive capacity manifests not only through biological pathways but also via behavioral adjustments—older adults may initially compensate through altered activity patterns or increased hydration, yet these strategies prove insufficient against persistent thermal stress. Such findings underscore the need for longitudinal monitoring frameworks that capture delayed and cumulative health impacts, particularly in populations with high baseline chronic disease burdens like China’s aging cohort.

Based on a time-dimensional analysis, our findings demonstrate that heat exposure impairs older adults’ self-rated health through both aggravated chronic diseases and weakened intergenerational support, thereby confirming the integrated biological-sociocultural pathways framework and supporting hypothesis H2 and H3. Physiologically, the increase of chronic diseases is an important mechanism by which long-term heat exposure weakens the self-rated health of older adults. We can find that in the case of long-term heat exposure, the deterioration of chronic diseases can account for 8.71% of the annual average-temperature’s impact on the decline in self-rated health among older adults, and it can also explain 21% of the damage to self-rated health in this demographic caused by the annual hot days. On the one hand, high temperature can increase the incidence of chronic diseases such as cardiovascular diseases and respiratory diseases among older adults. On the other hand, continuous heat exposure can place a greater physiological burden on older adults with chronic diseases, making it more difficult for them to adapt to climate extremes, leading to more chronic diseases (16, 21). In terms of family culture, long-term heat exposure weakens children’s economic and emotional support, which reduces the protective effect of children’s intergenerational support on self-rated health of older adults. Unlike Western systems that rely more on public health services (53), China’s unique “filial piety culture” makes health security for older adults more dependent on the financial and emotional support of their children (54, 55). Our research found that under long-term heat exposure, the mediating effect of care support is relatively limited, while economic support and emotional support can, respectively, explain 24.35 and 19.3% of the annual hot days’ damage to the self-rated health of older adults. This suggests that high-temperature environments can break the original health barriers of older adults, which is manifested in the following two aspects. On the one hand, high temperature may affect children’s support ability. Studies have found that heat exposure may lead to workers’ work efficiency decrease, reduced income and health deterioration (56). On the other hand, in a high temperature environment, emotional disorders are prone to occur in summer. Moreover, the interaction between older adults and their children may be reduced due to factors such as home isolation or physical discomfort, which may lead to a decline in emotional support. This mechanism differs from the stable support network model proposed by Luo et al. (57), where we suggest that climatic stress factors may disrupt the traditional kinship structure. Our research indicates that, in addition to biomedical pathways (12), high temperatures can also exacerbate health risks among older adults through culturally embedded social systems.

This study has several limitations. First, although the self-rated health used in this study is simple and feasible, its limitation as a subjective measurement tool is that it is susceptible to individual cognition and cultural background, which may weaken its cross-population comparability and the accuracy of reflecting changes in objective health status. Second, unmeasured confounding factors, such as air conditioning usage or nearby green spaces, which may modulate the relationship between heat exposure and health. Third, these mechanistic pathways fail to account for interventions at the government level. Beyond individual and household pathways, governments also play a critical role in addressing climate change. For instance, they impleme21nt health and public health policies, as well as comprehensive interventions targeting key regions and sectors to adapt to climate change through resource allocation and cross-departmental collaboration. Finally, the study does not thoroughly explore how regional climatic differences affect the health of older adults. The dataset does not cover all provinces in China, limiting the ability to fully capture regional variations in climate change impacts. Therefore, future research should investigate how regional climatic differences influence the health of older adults, considering factors such as socioeconomic development and local healthcare conditions. This will help identify vulnerable regions and populations, enabling the formulation of effective climate change adaptation policies.

## 5 Conclusion

This study redefines thermal vulnerability as a time-layered, culturally mediated phenomenon requiring multisectoral solutions. By demonstrating how China’s aging trajectory intersects with climate warming through both biomedical and social relational pathways, we provide a framework for other nationals facing similar dual challenges. The proposed interventions—individual chronic disease management (58), household support reinforcement (24), offers a blueprint for climate-resilient healthy aging policies (59). As global temperatures rise, protecting vulnerable older adults demands not only technological solutions but also the preservation of cultural protective factors increasingly threatened by environmental change.

## Data Availability

The datasets presented in this study can be found in online repositories. The names of the repository/repositories and accession number(s) can be found at: https://opendata.pku.edu.cn/dataverse/CHADS.

## Footnotes

^1^ https://opendata.pku.edu.cn/dataverse/CHADS
