# Supplementary material for: Heat exposure and self-rated health in older Chinese adults: the mediating roles of chronic disease and intergenerational support, 2008–2018 CLHLS
Source: Front Public Health. 2025 Sep 25;13:1636724. doi: 10.3389/fpubh.2025.1636724 (PMC12507916; doi:10.3389/fpubh.2025.1636724)
Supplement: Supplementary file 1 [file Image_1.pdf]

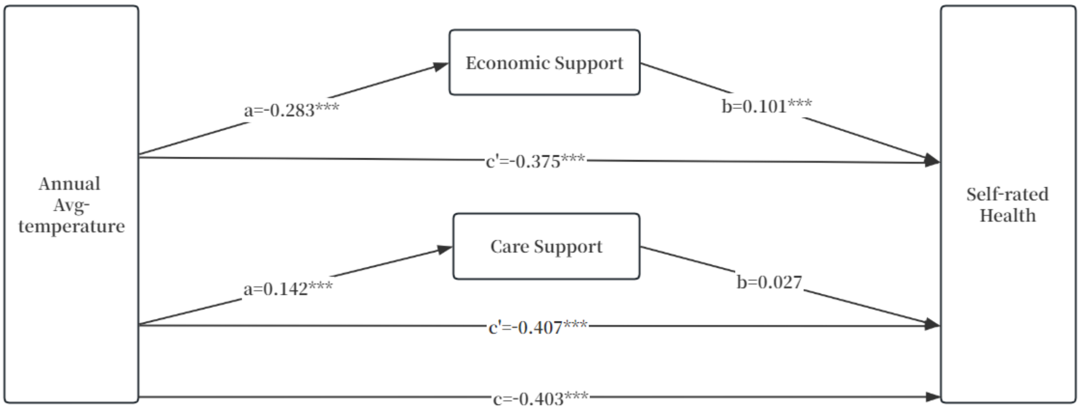

Note: ① * $p < 0.1$, ** $p < 0.05$, *** $p < 0.01$； ② Control variables: Residence, Age, Co-residence type, Living standards, Marital status and ADL. a b c c' is the path coefficient in the mediating effect analysis of the three-step method.

Supplementary Figure 1. Triangulation of the mediating effect of economic support and care support on the effect of annual average-temperature on self-rated health of the elderly.
