# Supplementary material for: Heat exposure and self-rated health in older Chinese adults: the mediating roles of chronic disease and intergenerational support, 2008–2018 CLHLS
Source: Front Public Health. 2025 Sep 25;13:1636724. doi: 10.3389/fpubh.2025.1636724 (PMC12507916; doi:10.3389/fpubh.2025.1636724)
Supplement: Supplementary file 2 [file Table_1.pdf]

Supplementary Table 1

Test of individual-level mechanism（N=9760）

	Chronic disease			
	M1	M2	M3	M4
**Short-term Heat Exposure**				
Daily Max-temperature	-0.002			
	(0.016)			
Heat Index		-0.036**		
		(0.016)		
**Long-term Heat Exposure**				
Annual Avg-temperature			0.260**	
			(0.125)	
Annual Hot Days				0.042***
				(0.016)
Control_	YES	YES	YES	YES
Constant	0.427***	0.428***	0.446***	0.452***
	(0.075)	(0.075)	(0.076)	(0.076)

Note：①Standard errors are in parentheses；②* $p < 0.1$, ** $p < 0.05$, *** $p < 0.01$；③Control variables: Residence, Age, Co-residence type, Living standards, Marital status and ADL.
