# Supplementary material for: Heat exposure and self-rated health in older Chinese adults: the mediating roles of chronic disease and intergenerational support, 2008–2018 CLHLS
Source: Front Public Health. 2025 Sep 25;13:1636724. doi: 10.3389/fpubh.2025.1636724 (PMC12507916; doi:10.3389/fpubh.2025.1636724)
Supplement: Supplementary file 3 [file Table_2.pdf]

Supplementary Table 2

Testing of the household-level mechanism（N=9760）

	Economic support				Care support	
	M1	M2	M3	M4	M5	M6
**Short-term Heat Exposure**						
Daily Max-temperature	0.007				0.007	
	(0.005)				(0.005)	
Heat Index		0.009*				0.002
		(0.005)				(0.004)
**Long-term Heat Exposure**						
Annual Avg-temperature			-0.283***			
			(0.040)			
Annual Hot Days				-0.052***		
				(0.005)		
Control_	YES	YES	YES	YES	YES	YES
Constant	0.697***	0.696***	0.676***	0.666***	-0.309***	-0.310***
	(0.024)	(0.024)	(0.024)	(0.024)	(0.021)	(0.021)
	**Care support**			**Emotional support**		
	M7	M8	M9	M10	M11	M12
**Short-term Heat Exposure**						
Daily Max-temperature			0.001			
			(0.005)			
Heat Index				-0.001		
				(0.005)		
**Long-term Heat Exposure**						
Annual Avg-temperature	0.142***				0.020	
	(0.035)				(0.037)	
Annual Hot Days		0.045***				-0.023***
		(0.005)				(0.005)
Control_	YES	YES	YES	YES	YES	YES
Constant	-0.299***	-0.283***	0.515***	0.515***	0.516***	0.502***
	(0.021)	(0.021)	(0.022)	(0.022)	(0.023)	(0.023)

Note：①Standard errors are in parentheses；②* $p < 0.1$, ** $p < 0.05$, *** $p < 0.01$；③Control variables: Residence, Age, Co-residence type, Living standards, Marital status and ADL.
